# Acute Anemia and Myocardial Infarction

**DOI:** 10.7759/cureus.17096

**Published:** 2021-08-11

**Authors:** Jaskamal Padda, Khizer Khalid, Gazala Hitawala, Nitya Batra, Sindhu Pokhriyal, Ayushi Mohan, Ayden Charlene Cooper, Gutteridge Jean-Charles

**Affiliations:** 1 Internal Medicine, JC Medical Center, Orlando, USA; 2 Internal Medicine, Avalon University School of Medicine, Willemstad, CUW; 3 Internal Medicine, Advent Health & Orlando Health Hospital, Orlando, USA

**Keywords:** anemia and mi, pathophysiology of anemia in mi, anemia and pci, treatment of anemia in mi, epidemiology of anemia

## Abstract

Various studies have established the prognosis of anemia in myocardial infarction (MI). Both chronic and acute anemia lead to poor outcomes in MI. Regardless, the association of anemia with MI and its management varies. In this study, the literature was analyzed to determine the association between acute anemia and MI based on the pathophysiology, outcomes, and management options. Acute anemia results in decreased blood supply and sudden hypoxia to the heart. Additionally, it exacerbates the preexisting compromised coronary blood supply in patients with MI. Thus, there is a disproportionate oxygen supply and demand ratio to the heart. It was found that anemia increases all-cause mortality in acute MI. However, it is unclear whether anemia is the direct contributor to mortality in these patients. For the management of MI, percutaneous coronary intervention (PCI) is commonly used. Increased incidence of hospital-acquired anemia (HAA) is reported in patients after PCI. However, the cause of HAA in these patients is not well established. Antiplatelet therapy in these patients is also considered to be the culprit for HAA. Nonetheless, no clear evidence is available. There is no consensus or criteria for the treatment of acute anemia in MI patients. Researchers have explored management options such as blood transfusion, erythropoietin-stimulating agent, and iron therapy. Further studies are warranted for a better understanding and management of MI in patients with anemia and vice versa.

## Introduction and background

Approximately 1.5 million people in the United States suffer from myocardial infarction (MI) annually, with a yearly incidence of 600 cases per 100,000 individuals. Coronary artery disease (CAD) is the leading cause of mortality in the United States, with about 500,000-700,000 deaths attributed to CAD every year [1]. It contributes to one-third of total deaths in adults older than 35 years of age [1]. Many risk factors have been established such as anemia, which has been well recognized in the prognosis of MI [2-4]. Anemia before admission, during hospitalization, and after MI has been associated with poor outcomes in patients [5].

Chronic anemia has a prevalence of 11-40% in patients with acute coronary syndrome (ACS) [6-8]. It is usually associated with older age and other comorbidities such as chronic kidney disease, hypertension, diabetes mellitus, peripheral vascular disease, and previous history of MI [8]. Additionally, an acute form of hospital-acquired anemia (HAA) is also common in MI patients with prevalence ranging from 30% to 60% and is associated with an increased risk of bleeding and hemodilution [9,10]. Treatment of MI utilizing thrombolytic, antithrombotic, antiplatelet therapy, and coronary revascularization procedures increase the risk of bleeding episodes which can precipitate anemia [11,12]. Intravenous fluids, medications, and fluid retention in acute heart failure caused by MI have been associated with hemodilution which can also contribute to the development of HAA [4]. Finally, anemia due to inflammation induced by ischemic myocardium can lead to an acute drop in hemoglobin (Hb) by 2-3 g/dL in one to two days. This is mediated by tumor necrosis factor-alpha and suppressed erythropoiesis [13].

Anemia in MI is speculated to cause an increase in catecholamines, a disturbance in oxygen supply and demand, the impairment of vascular healing, the depletion of nitric oxide and reactive oxygen species-mediated ischemia-reperfusion injury, and pathological ventricular remodeling [8,14-16]. All of these factors interact and lead to unfortunate outcomes such as heart failure, arrhythmias, risk of bleeding, and higher mortality compared to nonanemic patients [5,9]. Many studies have shown that clinicians tend to under-prescribe standardized treatment of MI such as antiplatelet drugs and percutaneous coronary interventions (PCIs) to anemic patients due to bleeding concerns [8,17]. Blood transfusions have not been consistently efficacious, and both liberal and restrictive transfusion strategies remain gray areas. The American Association of Blood Banks has no recommendations regarding transfusions for ACS patients who are hemodynamically stable in the hospital [18]. Furthermore, some studies have described chronic anemia and acute HAA as different entities with varying effects on prognosis in patients with MI [9,10]. These issues warrant further investigation to better understand and manage MI in patients with anemia.

## Review

Myocardial infarction

**Figure 1:**
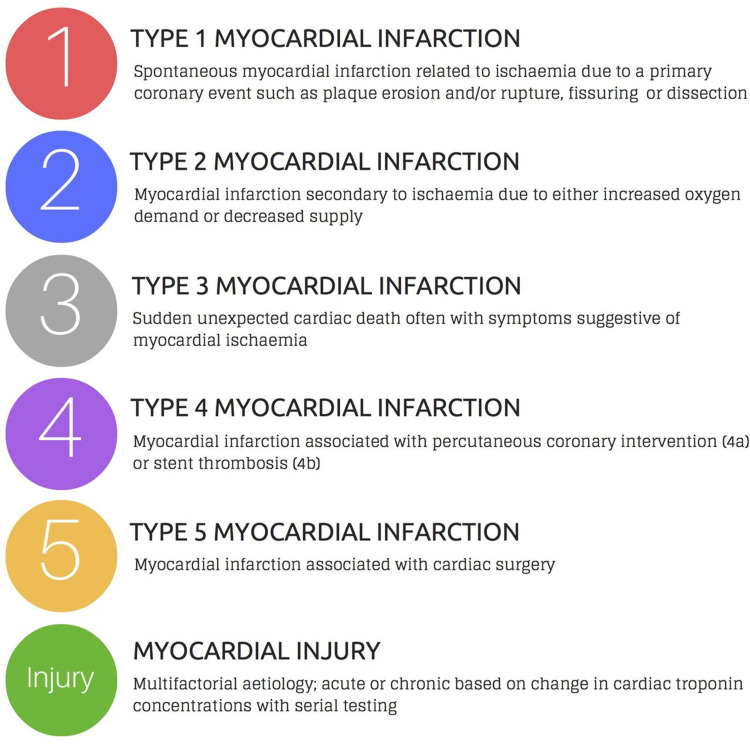
Subtypes of myocardial infarction. Copyright/License: This figure is from an open-access article distributed under the terms and conditions of the Creative Commons Attribution (CC BY 4.0) license (http://creativecommons.org/licenses/by/4.0/). No modifications were made to the original figure. Chapman AR, Adamson PD, Mills NL: Assessment and classification of patients with myocardial injury and infarction in clinical practice. Heart. 2016, 103:10-8. 10.1136/heartjnl-2016-309530 [21].

MI is the term used to describe myocyte cell death caused by reduced blood flow, resulting in ischemia and decreased perfusion to a portion of the heart. It can occur with or without symptoms and is typically diagnosed using an electrocardiogram (ECG), elevated biomarkers, or cardiac imaging [19,20]. MI affects about 1.5 million individuals in the United States annually, with a yearly incidence of 600 cases per 100,000 individuals. It presents more commonly in black patients compared to white, and the mortality rate is about three times higher in men than women [1]. Figure 1 shows the classification of the subtypes of MI [21].

Previously thought to be caused by a clot within an epicardial artery, it is now well established that MI is caused by ischemia resulting from a perfusion imbalance between supply and demand [19,20]. For the organs in our body to function adequately, blood supply should satisfy the required oxygen demand, which is referred to as the supply-demand ratio. An imbalance in this ratio concerning the heart can result from tachycardia (due to increased demand) or decreased blood pressure (caused by decreased supply), resulting in ischemia. Table 1 lists the possible causes of ischemia resulting in MI [19].

**Table 1:** Possible causes of ischemia that can lead to myocardial infarction. MI: myocardial infarction; CAD: coronary artery disease. Adopted from Saleh and Ambrose [19].

Ischemic condition that can result in MI	Causes
Imbalance of supply-demand ratio	Decreased supply or increased demand
CAD	Atherosclerosis
Coronary artery embolization	Atrial fibrillation, ventricular aneurysms, prosthetic valves, infected native heart valves
Systemic hypotension	Shock, anemia, tachyarrhythmias, hyperthyroidism, epicardial coronary artery stenosis
Spontaneous coronary artery dissection	-
Coronary spasm	Idiopathic, drug-induced
Takayasu’s arteritis	-
Giant cell arteritis	-

**Figure 2:**
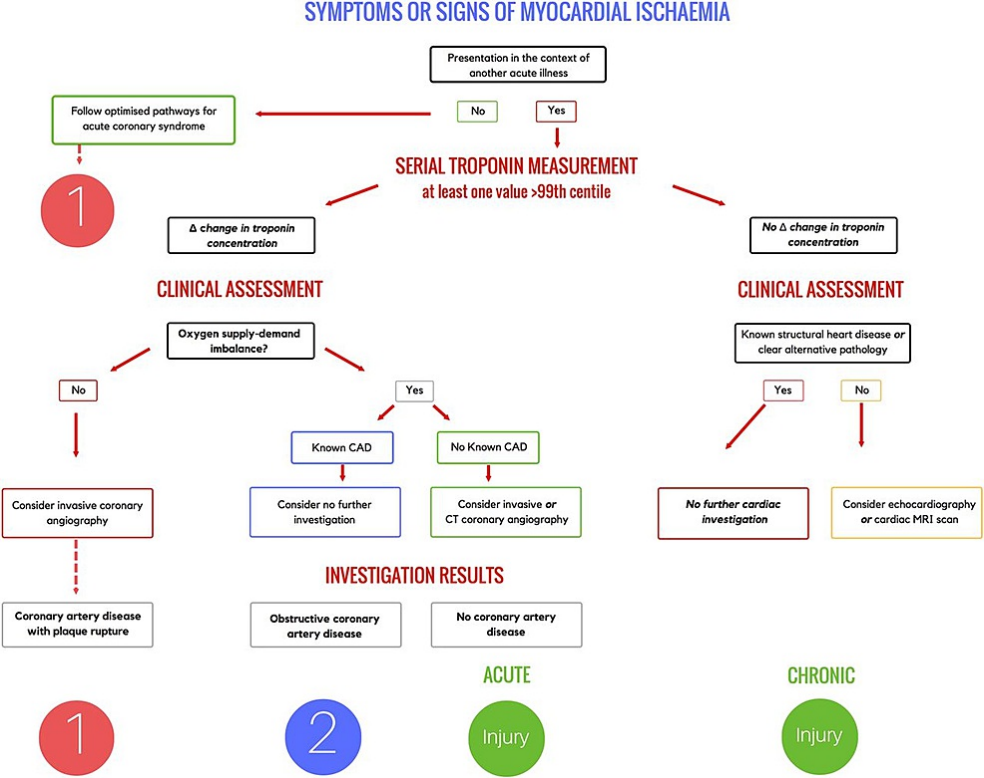
Diagnosis of myocardial infarction. MRI: magnetic resonance imaging; CT: computed tomography; CAD: coronary artery disease Copyright/License: This figure is from an open-access article distributed under the terms and conditions of the Creative Commons Attribution (CC BY 4.0) license (http://creativecommons.org/licenses/by/4.0/). No modifications were made to the original figure. Chapman AR, Adamson PD, Mills NL: Assessment and classification of patients with myocardial injury and infarction in clinical practice. Heart. 2016, 103:10-8. 10.1136/heartjnl-2016-309530 [21].

Ischemia is typically diagnosed in patients using their history and ECG findings. Possible presenting symptoms include chest pain, pain radiating to the upper extremity and/or jaw, epigastric discomfort, dyspnea, nausea, diaphoresis, or syncope. This discomfort generally lasts at least 20 minutes. It becomes more difficult to diagnose MI because these symptoms can also be caused by disorders in other organ systems such as neurological, musculoskeletal, gastrointestinal, or pulmonary [20]. MI diagnostic criteria consist of a rise/fall or both of specific cardiac biomarkers, with troponin being the most preferred biomarker. At least one value should be above the 99th percentile of the upper reference limit in addition to other evidence of MI. Some of the findings include cardiac wall motion abnormalities, the indication of a thrombus, and ECG changes such as ST-segment changes, presence of Q-waves, and new left bundle branch block [19,22]. Table 2 lists the imaging techniques used to diagnose MI along with their function [20]. Additionally, the step-by-step pathway to diagnose MI is explained in Figure 2 [21].

**Table 2:** Imaging techniques used to detect myocardial infarction. MRI: magnetic resonance imaging; CT: computed tomography. Adopted from Thygesen et al. [20].

Imaging technique	Function
Echocardiography	Assessment of myocardial thickness, thickening, and motion at rest
Radionuclide imaging	Detects areas of infarction and perfusion abnormalities
MRI	Assessment of myocardial function
Contrast-enhanced CT	Detects areas of infarction

The current management of ST-elevation myocardial infarction (STEMI) is PCI if the hospital is capable of performing the technique. Alternatively, thrombolytic drugs are administered before the patient can be taken to a hospital where angiography and/or PCI can be performed. These procedures are necessary to restore blood flow and reduce long-term complications. A stent may be needed to maximize the opening of the artery. Physical damage to the heart such as papillary muscle rupture with severe mitral regurgitation or rupture of the interventricular septum requires emergent surgery. In patients with triple-vessel disease or disease of the left main artery, coronary artery bypass graft surgery should be considered depending on severity. Management of non-ST-elevated MI differs from STEMI. High-risk patients undergo invasive treatment such as angiography, PCI, and/or coronary artery bypass graft, along with medical therapy. Low-risk patients are managed conservatively with medical therapy [19]. Medical therapy that is routinely used in MI includes antiplatelet drugs (aspirin, clopidogrel), anticoagulants (heparin or bivalirudin), nitroglycerin, beta-blockers, angiotensin-converting enzyme inhibitors, and statins [23].

Epidemiology of anemia

**Figure 3:**
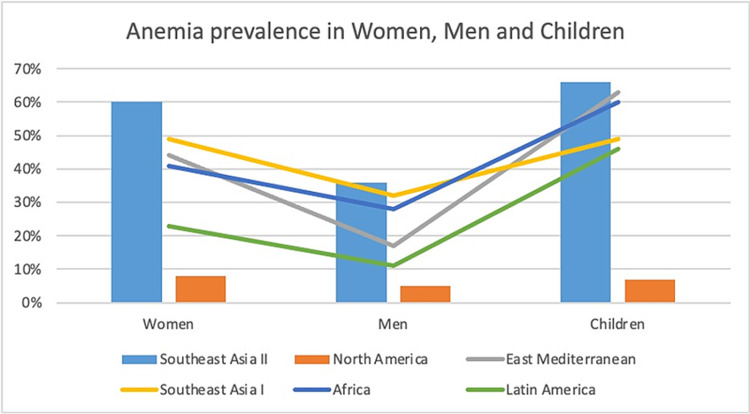
Anemia prevalence across the globe. Southeast Asia I: Indonesia, Sri Lanka, and Thailand [28]. Southeast Asia II: Bangladesh, Bhutan, Democratic People’s Republic of Korea, India, Maldives, Myanmar, and Nepal. North America: Including Cuba. Africa: Excluding Egypt, Morocco, Somalia, Sudan, and Tunisia. East Mediterranean: Afghanistan, Djibouti, Egypt, Iraq, Morocco, Pakistan, Somalia, Sudan, and Yemen. Latin America: Excluding Cuba. The image was created by one of the authors (Ayushi Mohan).

Anemia is a considerable public health condition. In 2019, anemia accounted for 58.6 million years of disability [24]. A study conducted by the World Health Organization (1993-2005) revealed that anemia affected 24.8% of the population across the globe, which was approximately 1.62 billion people [25]. Another study showed that the burden of anemia affected approximately one-third (32.9%) of the world in 2010, with pregnant women being affected the most (46%), followed by children less than five years of age (42%) and reproductive age group females (39%) [26]. On the other hand, a cohort study conducted on 435 hospitalized patients in internal medicine revealed a similar prevalence of anemia in both males and females. The study also showed that anemia was more prevalent in patients admitted for infections compared to other causes [27]. Geographically, Africa and South Asia topped the list for the prevalence of anemia [24,25]. Figure 3 shows the prevalence of anemia across the globe [28].

Anemia can be acute or chronic. Acute anemia can be caused by acute blood loss and hemolysis, while chronic anemia is preceded by chronic blood loss and defective red blood cell (RBC) formation [26]. Among various causes of reduced Hb, iron deficiency contributes to approximately half of the total number of affected people [25]. However, the cause of mild anemia is unknown in a significant number of elderly patients and can be myelodysplastic syndrome in some patients [28].

The prevalence of anemia in ACS ranges between 10% and 43% [29]. Multiple risk factors such as Hb disorders and nutritional deficiencies, specific to particular populations, account for this difference. Undoubtedly, the diagnosis of anemia in MI patients, both before and after hospitalization, is associated with a worse prognosis [29]. A study in Hamadan Ekbatan Hospital (2012-2013) involving 320 MI patients revealed a 19.1% prevalence of anemia, of which 83.6% of the patients died [30]. Even though the prevalence and incidence of anemia are significant, it is often overlooked, particularly in older patients with multiple other comorbidities [27].

Pathophysiology of anemia in myocardial infarction

In a retrospective cohort study involving 422,855 patients in the United Kingdom, one in four patients presenting with ACS had anemia. Anemia was also associated with adverse hospital outcomes in these patients [8]. It is well established that anemia has a multifold influence on distinct body systems. Corresponding to the other organs in the body, the heart demands oxygen supply through blood. The heart receives approximately 4-6% of the cardiac output [31]. The oxygen delivery to the tissue is measured as a product of cardiac output and arterial oxygen content. Thus, the determinants of decreased oxygen delivery to the tissues are decreased Hb concentration resulting in anemic hypoxia, decreased cardiac output causing stagnant hypoxia, or decreased Hb saturation resulting in hypoxic hypoxia [32,33].

The sequel of acute anemia causes decreased circulating erythrocyte mass, Hb, and hematocrit. Furthermore, it also causes tachycardia which shortens the diastolic phase of the cardiac cycle and decreases arterial pressure [34]. Acute anemia results in decreased blood flow (stagnant hypoxia) and oxygen-carrying capacity (anemic hypoxia). To preserve oxygen supply to the tissue, the heart begins to work vigorously. Consequently, there is increased oxygen requirement by the heart [35]. The myocardium consumes almost 60-75% of the oxygen delivered to the heart [36]. Furthermore, left ventricular perfusion relies on the diastolic phase of the cardiac cycle. The oxygen delivery to the heart depends on the coronary blood flow. However, in patients with MI, the blood supply to the heart is compromised [35].

In MI patients, in association with acute anemia (e.g., acute blood loss), there is decreased venous return to the heart which increases the oxygen requirement because of the additional work done by the heart. Moreover, as mentioned earlier, acute anemia results in tachycardia which shortens the diastole and further decreases the blood supply to the heart [37]. Typically, the decreased arterial oxygen content is compensated by increasing the coronary flow through vasodilation. However, this compensatory mechanism fails in a heart with decreased functional coronary reserve capacity [38,39]. Additionally, in acute normovolemic anemia, the left ventricular oxygen consumption is maintained by increasing oxygen extraction from the coronary blood. In contrast, in a similar scenario, the coronary reserve is compromised at hematocrit values that are half of normal, especially in coronary occlusive diseases [40].

Anemia and poor outcomes in myocardial infarction

The effect of anemia in patients with MI has long been a subject of interest. Anemia has been projected as a poor prognostic factor in both short- and long-term outcomes after MI [41,42]. Anemic patients are more likely to have concomitant risk factors such as hypertension, hypothyroidism, rheumatological diseases, chronic kidney disease, malignancy, and congestive heart failure, as mentioned previously [43]. The presence of these comorbidities does not just make MI a more challenging condition to manage, but they increase the risk of complications in this subset of patients, thus affecting their short- and long-term outcomes [3,44]. It is also well known that anemic patients are more likely to present with congestive cardiac failure and cardiogenic shock following MI, which is associated with increased morbidity as well as mortality in these patients [44,45]. Furthermore, anemia has been associated with an exaggerated inflammatory state which has been implicated in impaired vascular healing in addition to causing hypoxic injury to the already damaged myocardium. This impairment in favorable myocardial remodeling after MI has been hypothesized as another possible mechanism by which anemia causes poor outcomes in MI patients [45-47].

In a study by Colombo et al. on 2,011 patients, those with acute MI who survived longer than 28 days were followed up for a median of 4.2 years. Possible differences in survival between anemic and nonanemic patients with acute MI were tested using Kaplan-Meier plots as well as log-rank tests. Following an initial unadjusted analysis, a multivariate adjustment for variables such as sex, age, body mass index, smoking status, history of angina or diabetes, acute MI type, left ventricular ejection fraction, and estimated glomerular filtration rate was done. They found that both mild and moderate-to-severe anemia was associated with a statistically significant increase in all-cause mortality, which attenuated after the multivariate analysis but remained statistically significant [48].

In another study, Moghaddam, et al. studied hospital outcomes in 1,919 STEMI patients, of which 322 patients had anemia. They measured several outcomes that were statistically significant such as length of hospital stay (p < 0.001), cardiac arrest (p = 0.022), heart failure (p = 0.003), and cardiogenic shock (p < 0.001). While their initial analysis revealed an increase in the all-cause mortality rate in these patients, after adjusting for multivariate variables such as infarct, location, and reperfusion times which can independently affect mortality, they found no association between anemia and mortality rates. Therefore, they concluded that although anemia may be a marker of increased mortality in patients with ACS, it is not clear if it directly increases mortality [49].

Furthermore, many studies have assessed the risk of bleeding in patients with MI and suggested that anemia is associated with higher blood loss in patients with MI. Mehta et al. and Manoukian et al. demonstrated an increased incidence of peri-PCI hemorrhagic complications in MI patients with anemia [50,51]. Additionally, Moghaddam et al. concluded that of the 322 anemic patients, the risk of major bleeding was 18.2% in anemic patients as opposed to 9.4% in those who did not have anemia [50]. The REPLACE 1 and 2 trials conducted by Nikolsky et al., which worked toward developing a prognostic risk score using several clinical and procedural variables, validated anemia as one of the predictive variables of major bleeding post-PCI in patients with MI undergoing interventional procedures [52].

Anemia and poor outcomes in percutaneous coronary intervention

A successful, timely, and sustained revascularization, commonly in the form of PCI, is the *sine qua non* of ACS treatment. Patients who develop acute HAA after this procedure have poor outcomes [9,10,53-55]. The definition of HAA was variable in different studies with some utilizing the WHO anemia threshold levels of 130 g/L in men and 120 g/L in women and some used age, gender, and race-specific criteria described by Beutler and Waalen [9,10]. A retrospective study was conducted in 2019 on 4,083 ACS patients who underwent PCI. Overall, 46.4% of patients had anemia at the time of discharge, of which 30.7% had newly developed anemia. These patients had an increased one-year risk of experiencing major cardiovascular adverse events (mortality, MI, and stroke) compared to nonanemic patients with a hazard ratio of 1.72 [9].

Another study in 2011 which used the Health Facts database to study 17,676 patients in the United States determined that 54.8% of patients had PCI, of whom 56.6% developed HAA. Hospital mortality of HAA patients was higher and increased with severity of anemia, with 3.9% in mild anemia (Hb: >11 g/dL), 8.1% in moderate (Hb: 9-11 g/dL), and 18.3% in severe HAA (Hb: <9 g/dL) [10]. On similar lines, in a prospective Translational Research Investigating Underlying disparities in acute MI Patients’ Health Status registry, 4,350 patients with acute MI were enrolled and 530 developed HAA, of whom 344 underwent PCI. At a 36-month follow-up, 31% of patients had persistent HAA and had a significantly higher mortality rate with a hazard ratio of 2.08 and significantly lower health status [53].

Another study in 2009 enrolling 2,909 patients with 74% patients undergoing PCI had an HAA rate of 46.8% and was associated with a higher mortality rate in moderate-to-severe anemia with a higher frequency of bleeding [54]. A research paper in 2009 that studied post-PCI anemia (defined as Hb <10g/dL) reported that 8.8% of patients developed post-PCI anemia, which was associated with a greater incidence of death at six months (6.5% vs. 1.7%), six-month major adverse cardiovascular event (recurrent MI or target vessel revascularization; 27.3% vs. 14.5%), and long-term mortality (25.8% vs. 8.7%) [55]. Due to various poor outcomes associated with anemia, some studies have found it to be a helpful risk stratification criterion in addition to conventional scoring systems such as Global Registry of Acute Coronary Events and Primary Angioplasty in MI [56-58].

Several mechanisms have been implicated to explain the causation of acute HAA in patients who undergo PCI. Procedural risk of the intervention and concomitant use of antiplatelet and antithrombotic medications increase the risk of bleeding, with dual antiplatelet therapy conferring an even higher risk [11,12]. Although bleeding does not entirely explain HAA, a study reported bleeding in 178 out of 1,321 patients with HAA. This study also correlated other factors such as age, female sex, white race, chronic kidney disease, STEMI, acute renal failure, use of glycoprotein IIb/IIIa inhibitors, in-hospital complications (cardiogenic shock), and length of stay with HAA [56]. Patients with HAA in one study had high grades of Killip Classification suggesting that hemodilution is also responsible for anemia [4]. Additionally, one study reported higher C-reactive protein levels in MI patients who had anemia indicating that chronic inflammation and cytokines such as transforming growth factor-beta may play a role in suppressing erythropoiesis and leading to anemia [9,13]. Studies analyzing HAA and its effects on MI are limited. Additional research and interventional studies are required to get further insights regarding HAA and its causes, effects, recognition, prevention, and treatment [9].

Interventions for the treatment of anemia in myocardial infarction

Three main modalities are considered for the management of MI patients with anemia include RBC transfusion, erythropoietin-stimulating agents (ESAs), and iron supplementation [59]. A study concluded that STEMI patients with coexisting anemia were associated with not receiving the American Heart Association (AHA)-recommended standard treatment and increased mortality. However, higher mortality could not be fully explained by the treatment difference between anemic and nonanemic STEMI patients [47]. Randomized control trials reveal that transfusing patients with Hb levels between 9 g/dL and 10 g/dL shows no advantage over transfusing patients with Hb levels between 7 g/dL and 8 g/dL [60]. Another study revealed that transfusion is not beneficial and may end up harming patients with cardiovascular disease or Hb levels of more than 10 g/dL [59]. Theoretically, transfusion increases oxygenation of organs and, in turn, the myocardium which should result in the reduction of ischemia; however, studies report contrary findings. Multiple reasons have been proposed for this including increased blood viscosity and systemic inflammation. Transfusion remains a vital therapy in the setting of acute anemia, with the Hb cut-off being debatable. Various studies show that a restrictive transfusion strategy leads to favorable outcomes compared to a more liberal strategy [29]. The American Association of Blood Banks published transfusion threshold guidelines in 2016 which recommend restrictive strategies but exclude ACS patients [29].

Although ESAs can also conceptually help in hypoxia, studies fail to show any benefit in patients with ACS. In fact, one study showed increased thrombotic complications like MI and stroke with ESAs [60]. Clinical trials performed on animals show a cytoprotective effect after administration of ESAs whereas clinical studies on chronic heart failure patients failed to produce a similar result. Side effects reported with ESAs include hypertension, venous thromboembolism, and increased mortality, particularly in patients with underlying chronic kidney disease [29,61]. There is inconsistent evidence of ESAs showing health benefits such as increased exercise tolerance and duration [61].

Iron is another alternative to RBC transfusion. A study showed improved New York Heart Association class in chronic heart failure patients with anemia who were supplemented with intravenous iron therapy. This led to improved quality of life and reduced cardiovascular events. Side effects such as oxidative stress and infection have been reported with iron therapy [29]. There is no standard protocol available for the treatment of iron deficiency anemia in ACS but AHA recommends giving priority to reducing the risks of bleeding in addition to the usual care [60]. Due to the large prevalence of anemia in MI patients and a substantial adverse prognosis associated with it, more studies are needed to establish a standard treatment algorithm.

## Conclusions

While MI has been a leading cause of mortality in the United States contributing to a significant number of deaths in younger individuals, anemia also has a significant global presence affecting the overall physical, psychological, as well as economic well-being of people globally. An association between the two which has long been a subject of both interest and speculation is now a more or less well-established phenomenon.

From this review article focusing on the impact of both chronic and acute anemia on the myocardium, it is more than clear that anemia has been well established as a marker of poor outcomes in patients with MI. Of the many studies conducted to analyze this relationship, controversies still exist, especially pertaining to the confounding factors in different studies levied upon multiple clinical variables which often coexist with either anemia or MI. Thus, more studies are needed to come to a clear conclusion regarding the exact effect of anemia on the myocardium as well as the outcomes.

Likewise, although blood transfusion, ESA, and iron replacement therapy have been suggested as possible treatment modalities in the management of anemia in MI patients, their optimal use and benefit in improving outcomes needs more studies. Seeing the magnitude of association of both these problems, it seems imperative to define clear treatment algorithms as well as clear thresholds for blood transfusions in patients of MI, irrespective of whether they undergo interventional procedures.
